# Familial Mediterranean fever in Armenian children with inflammatory bowel disease

**DOI:** 10.3389/fped.2023.1288523

**Published:** 2024-02-12

**Authors:** Gayane Amaryan, Tamara Sarkisian, Artashes Tadevosyan, Christian Braegger

**Affiliations:** ^1^ National Pediatrics Center for Familial Mediterranean Fever, “Arabkir” Medical Complex-Institute of Child and Adolescent Health, Yerevan, Armenia; ^2^ Department of Pediatrics, Yerevan State Medical University, Yerevan, Armenia; ^3^ Center of Medical Genetics and Primary Health Care, Yerevan, Armenia; ^4^ Department of Medical Genetics, Yerevan State Medical University, Yerevan, Armenia; ^5^ Department of Public Health and Health Care Organization, Yerevan State Medical University, Yerevan, Armenia; ^6^ Nutrition Research Unit, Children’s University Hospital in Zürich, Zürich, Switzerland

**Keywords:** children, inflammatory bowel disease, familial Mediterranean fever, clinical characteristics, genetic characteristics

## Abstract

Inflammatory bowel disease (IBD) and familial Mediterranean fever (FMF) are inflammatory diseases with complex interactions among genetic, immune, and environmental factors. FMF is a monogenic autoinflammatory disease, characterized by recurrent febrile attacks and polyserositis, and is manifested mainly in childhood. FMF is widespread in Armenia. There are reports on the concurrent occurrence of FMF and IBD. *MEFV* gene mutations may have a disease-modifying effect on IBD. We have investigated the frequency of *MEFV* mutations and FMF in Armenian children with IBD and their influence on the clinical course. A total of 69 untreated IBD patients under 18 years of age were enrolled: 52.1% (36) had ulcerative colitis (UC), 21.7% (15) had Crohn's disease (CD), and 26.0% (18) had unclassified colitis (IBD-U). The frequency of FMF among them was 36.2% (25/69), and *MEFV* mutations were identified in 53.6% (37/69). The highest rate of *MEFV* mutations and FMF was in UC patients (61.1% and 41.6% respectively). In all, 56.7% (21/37*)* of IBD patients with *MEFV* mutations had *M694V* mutated alleles, mainly in compound heterozygous and heterozygous states. There were no associations in the group of IBD patients with coexisting FMF (25), either between any *MEFV* mutation and type of IBD or coexistence of FMF. Overall, 36.0% (9/25) of them developed VEO IBD and carried mainly the *M694V* mutation. We concluded that the carrier frequency of *MEFV* mutations among Armenian pediatric IBD patients was rather high (53.6%), especially for UC. It was suggested that the MEFV gene is not necessarily a susceptibility gene but most likely modifies the course of IBD. *MEFV* genetic testing was recommended for Armenian pediatric IBD patients, especially for VEO UC and IBD-U, atypical IBD course, or resistance to the conventional treatment. They should also be asked for isolated febrile attacks, recurrent arthritis, and family history, even in the absence of FMF typical symptoms, to rule out FMF and its complications.

## 1 Introduction

Inflammatory bowel disease (IBD)—comprising ulcerative colitis (UC), Crohn's Disease (CD), and unclassified colitis (IBD-U)—is the most common form of chronic intestinal inflammation, which has a multifactorial etiology with complex interactions among genetic, immune, and environmental factors (1). Familial Mediterranean fever (FMF; ОMIM 249100) is another inflammatory disease as well. It is the most common autosomal recessively inherited monogenic autoinflammatory disease in the group of hereditary periodic fever syndromes (HPFS), mostly found in Sephardic Jews, Armenians, Turks, and Arabs (2, 3). It is characterized by recurrent attacks of fever and painful aseptic peritonitis, pleuritis, synovitis, elevated acute phase of reactants, and its most severe complication—secondary amyloidosis (4–7). Being an ethnic disease, FMF is widespread in Armenia, with a high rate of *MEFV* mutation carriers of 1:3–4 (0.21) and a marked FMF prevalence of 54.7 per 10,000 of the total population (8–15). FMF manifests mainly in childhood and often has an early or atypical onset.

The concomitant presence of *MEFV* mutations in diseases other than FMF may modify their presentation and severity. IBD and FMF are inflammatory disorders sharing some common clinical features such as recurrent abdominal pain, diarrhea, fever, and arthralgia/arthritis, etc. UC and FMF are both characterized by a recurrent pattern of presentation with periods of remission and flares associated with neutrophilic infiltration at the site of injury and dysregulation of apoptosis (16, 17). The *MEFV* gene, responsible for FMF, is involved in inflammatory reactions through altered leukocyte apoptosis, secretion of interleukin-1beta (IL-1b), activation of the NF-kappa B pathway, and, thereby, the degree of inflammation. IBD appears to be more frequent in FMF-matched ethnic populations. There are reports on the concurrent occurrence of FMF and IBD. As known, the level of awareness of FMF is far from being sufficient, and it is assumed that there may be many patients with FMF who are under observation without an accurate diagnosis (17–20). The possibility of developing intestinal vasculitis in FMF is also suggested (21, 22).

Despite the presence of sufficient data on the possible link between the pathogenesis of IBD and FMF, the question of whether UC and CD co-exist or are associated with FMF remains open. It is known that susceptible CD loci localize to chromosome 16q and contain the NOD2-CARD15 CD susceptibility gene. On the other hand, the gene responsible for FMF, *MEFV*, is in chromosome 16p13 encoding for pyrin, which has been linked to the apoptotic cascade through the caspase recruitment domain (CARD). It is suggested that the NOD2/CARD15 gene product belongs to the same superfamily of death domain protein. In practice, the concurrent occurrence of FMF and CD remains to be demonstrated. *MEFV* mutations are not associated with Crohn's disease susceptibility, yet the presence of these mutations appears to be associated with a structuring disease pattern, and extraintestinal disease *MEFV* mutations may have a disease-modifying effect on IBD (18, 19, 23, 24).

On the other hand, studies on the concordance of FMF and IBD, especially with VEO, have suggested that there is a relationship between IBD and FMF (24–27). Several studies have shown that patients with a diverse spectrum of rare genetic disorders can present with inflammatory bowel disease (monogenic IBD). Patients with these disorders often develop symptoms during infancy or early childhood, along with endoscopic or histological features of Crohn's disease, ulcerative colitis, or IBD unclassified. Defects in interleukin-10 signaling have a Mendelian inheritance pattern with complete penetrance of intestinal inflammation. Several genetic defects that disturb intestinal epithelial barrier function or affect innate and adaptive immune function have incomplete penetrance of the IBD-like phenotype. Due to the broad spectrum of these extremely rare diseases, a correct diagnosis is frequently a challenge and often delayed. In many cases, these diseases cannot be categorized based on standard histological and immunologic features of IBD. Genetic analysis is required to identify the cause of the disorder and offer the patient appropriate treatment options (26, 28–30)

## 2 Material and methods

### 2.1 Study setting and data collection methods

The aim of this study was to investigate the frequency of MEFV gene mutations and FMF disease in Armenian children with IBD and their influence on the IBD clinical course.

The objectives were the following: (1) to evaluate the spectrum of MEFV gene mutations and genotypes in Armenian children with IBD; (2) to find out the possible association between MEFV genotypes and the type of IBD and their influence on the IBD clinical course.

The cohort of patients with confirmed IBD patients were hospitalized in the General Pediatrics Department of Arabkir MC- ICAH in 2014–2016 and followed up in the outpatient clinic of the pediatric gastroenterology service of the same hospital up to 2019. IBD diagnosis was determined according to the ESPGHAN Porto criteria (endoscopic, radiologic, and histologic) (31). The inclusion criteria were the following: (1) age of IBD patients under 18 years;(2) the informed consent of parents to participate in the study. The exclusion criterion was refusal to participate in the study.

FMF diagnosis was confirmed in 25 (36.2%) of the 69 enrolled IBD patients based on the Tel Hashomer international criteria and genetic testing (32, 33). The molecular genetic analysis for 12 MEFV gene mutations, most common for Armenians, was performed for all the IBD patients at the Center of Medical Genetics and Primary Health Care of Armenia *(M694V, V726A, M680I (G/C), M680I (G/A), R761H, E148Q, F479l, M694I, K695R, P369S, K695R, I692del)* by reverse hybridization, restriction analysis, PCR, and sequencing (8–10). IBD patients with concomitant FMF were also followed up in the FMF outpatient clinic of NPC FMF of Arabkir MC—ICAH during the same period.

The patients and their medical charts were examined and reviewed to determine the demographic (age of IBD and/or FMF onset; age at IBD and/or FMF diagnosis; sex; consanguinity) and clinical characteristics of the IBD and FMF patients (symptoms and their duration, IBD type and location, extraintestinal manifestations, therapy approaches, duration of follow-up). The laboratory evaluations of IBD were based on the ESPGHAN conventional IBD diagnostic criteria: hemoglobin, erythrocyte sedimentation rate (ESR), C-reactive protein (CRP), thrombocyte count, ferritin, albumin level, and fecal calprotectin at diagnosis. The Pediatric UC Activity Index (PUCAI) and the Pediatric CD Activity Index (PCDAI) were used for evaluating the disease severity (34–36). Proteinuria was also checked during the follow-up (proteinuria of more than 1 g/day needs to exclude the presence of amyloidosis in renal biopsies).

The IBD patients were divided into two groups (with and without FMF) depending on the coexisting FMF disease. They were also distributed into the next two groups (with and without MEFV mutations) depending on the presence of the mutations. All the above-mentioned demographic, clinical, and laboratory parameters were compared between these groups of IBD patients.

### 2.2 Statistical analysis

After checking the normality of the distribution of variables by the Kolmogorov–Smirnov test, non-parametric statistical tests were applied. All the statistical analyses were performed using the software Biostat for Windows (Version 4.03) and SPSS 16.0. Fisher's exact test or chi-square test was used to compare the frequency of MEFV mutations among the UC, CD, and IBD-U patients and their clinical symptoms. The Mann–Whitney *U*-test was used for the analysis of some demographic and laboratory data of the IBD patients in the groups with/without *MEFV* gene mutations and with/without FMF. To compare the two medians, the Kruskal–Wallis test was used. *P* value <0.05 was always considered statistically significant. To describe the demographic and laboratory numerical data, routine descriptive statistics were used.

## 3 Results

### 3.1 Patients’ socio-demographic, clinical, and laboratory characteristics

The cohort of 69 patients with an IBD diagnosis confirmed by the international Porto criteria were hospitalized in the General Pediatrics Department of Arabkir MC- ICAH in 2014–2016 and followed up in the outpatient clinic of the pediatric gastroenterology service of the same hospital up to 2019. There were 45 male and 24 female patients with aged between 3 months and 18 years (mean age: 9.36 ± 0.62) at IBD diagnosis. UC was diagnosed in 36 of 69 (52.1%) IBD patients, CD in 15 (21.7%), and IBD-U in 18 children (26.0%).

All the patients were of Armenian nationality, representing all the regions of the country, and most of them lived in urban settlements. The median age of IBD patients was 10 years (range, 0.3–18) at IBD diagnosis. The male/female ratio was 1.87:1 (45 male and 24 female). The mean age of IBD onset was 9.13 ± 0.65 years (range, 0.3–18).

The diagnosis of both IBD and FMF diseases was performed relatively late (at 9 years and 7 years of age, respectively). In the majority of IBD patients with coexisting FMF, the diagnosis of FMF was confirmed after the IBD (19 of 25), which coincides with the data of other authors (36). The average duration of follow-up after diagnosis of IBD was 58.56 ± 7.44 months.

The IBD location was as follows: ileocolitis 12 (17.4%), colitis 22 (33.9%), and pancolitis 35 (50.7%). The UC patients (36) were evaluated with the PUCAI, and most of them (22) had moderate disease activity, 2 had severe, and 9 had mild disease (PUCAI median 45; range 10–65). All the CD patients (15) had severe disease activity (PCDAI median 50, range 30–80).

FMF was confirmed in 25 of 69 (36.2%) IBD patients. Depending on the IBD type, the frequency of FMF disease was as follows: in 15 of 36 (41.6%) UC patients, in 7 of 18 (38.8%) IBD-U, and in 3 of 15 (20.0%) CD. In 6 (8.6%) IBD patients, FMF was confirmed before IBD diagnosis due to the prior testing for *MEFV* mutations. Genetic analysis was conducted based on an identified history of recurrent episodes of fever with abdominal pain and/or chest pain and/or arthropathy and/or family history of FMF. Following the diagnosis of FMF, treatment with colchicine was started. The mean age of FMF diagnosis was 7.94 ± 1.13 years. The average duration of FMF follow-up was 74.28 ± 10.32 months.

In IBD patients with FMF, the localization site at IBD was as follows: most *UC* patients (12 of 15; 80%) had pancolitis, and 3 had colitis; 2 of 3 *CD* patients developed ileocolonic disease, and 1 had pancolitis; 5 of 7 *IBD-U* children had left-sided colitis, and 2 had pancolitis.

The UC patients with and without FMF had a moderate disease activity on the PUCAI (median 50 and 37.5, range 20–65 and 10–55, respectively) with no significant difference between the groups (*p* = 0.23; *by Kruskal–Wallis*). A total of 12 CD patients without FMF had severe activity on the PCDAI (median 50, range 30–80), while 3 CD patients with FMF also had severe activity on the PCDAI (40, 50 and 60 values).

The most frequent symptoms in IBD patients were hematochezia in 56 (81%) patients, abdominal pain or tenesmus in 48 (69.6%), and diarrhea in 45 (65.2%).

Extraintestinal manifestations were noticed in 12 (17.3%) IBD patients, mainly mono-oligoarthritis in 9 patients (13.0%). Perianal disease developed in 4 (5.8%) CD patients (two fistulae and two abscesses). There was no consanguinity. There were no patients with proteinuria either.

All IBD patients were untreated prior to hospitalization. After confirmation of IBD diagnosis, the appropriate treatment was started in the hospital. A total of 54 patients (78%) were treated with Salofalk, 35 (50.7%) with methylprednisolone, and 49 (71%) were receiving immune suppressive treatment with azathioprine.

Conventional colchicine therapy was used in all 25 IBD patients with coexisting FMF and FMF remission, and a positive response was achieved in 22 (88%) of them. The colchicine dosage was increased up to the maximum level in 2 UC patients and in 1 CD patient with a severe M694V homozygous and compound heterozygous genotypes because of a concomitant flare of FMF.

There was no statistical difference in IBD patients with or without FMF when comparing most of the demographic, clinical, and laboratory characteristics, including extraintestinal symptoms, exacerbation rates (*p* = 0.32), the number of IBD relapses (*p* = 0.2), and immunosuppressive therapy (*p* = 0.5) (Table 1). However, lower ferritin blood level (*p* = 0.05) and longer hematochezia (*p* = 0.026) were significantly more often observed in IBD patients with concomitant FMF, possibly due to the chronic immune inflammation. The possibility of developing intestinal vasculitis as an integral part of FMF is suggested by some authors due to the high frequency of occult blood detection in stool during attacks of FMF (37–39).

**Table 1:** Demographic, clinical, and laboratory characteristics of IBD patients with FMF (group I) and without FMF (group II).

Features	Group I (*n* = 25)	Group II (*n* = 44)	*P*-value
Gender ratio (M/F)	17/8	29/15	0.85^c^
Age at IBD diagnosis (year)	9.78 ± 1.22	9.14 ± 0.7	0.65^b^
Age at IBD onset (year)	9.16 ± 1.3	9.12 ± 0.72	0.9^b^
Follow-up for IBD (year)	6.19 ± 0.86	4.88 ± 0.62	0.86^b^
Clinical characteristics			
Disease type CD/UC/IBD-U (*n*)	3/15/7	12/22/11	0.4
Abdominal pain/tenesmus (mo)	7.35 ± 1.0	5.83 ± 0.99	0.31^b^
Hematochezia/hemocolitis (mo)	7.55 ± 1.16	4.75 ± 0.64	0.026^b^
Diarrhea (mo)	5.75 ± 0.92	4.67 ± 1.00	0.45^b^
Fever (mo)	2.82 ± 0.88	3.99 ± 0.65	0.066^b^
Median PUCAI for UC	50, range 20–65	37.5, range 10–55	0.23^d^
Median PCDAI for CD	3 patients: 40, 50, 60	50, range 30–80	
Extraintestinal manifestations (*n*)	6 (4 arthritis, 2 others)	6 (5 arthritis,1 other)	0.32^c^
Immunosuppressive treatment (*n*)	14 M/pred, 14 AZA	26 M/pred, 26 AZA	0. 94^c^
Relapses (number)	3.0 ± 0.36	2.32 ± 0.37	0.2^b^
Laboratory characteristics			
Hb (g/L) at diagnosis	111.5 ± 5.2	115 ± 3.24	0.56^b^
Ferritin (ng/ml) at diagnosis	17.11 ± 3.94	28.53 ± 3.40	0.05^b^
Albumin (g/L) at diagnosis	42.15 ± 1.16	41.51 ± 1.18	0.71^b^
ESR (mm/h) at diagnosis	22.8 ± 2.86	23.25 ± 2.99	0.93^b^
CRP (mg/ml) at diagnosis	28.05 ± 7.41	30.65 ± 10.01	0.64^b^
Platelets (10^9/L) at diagnosis	348.83 ± 4.26	368.75 ± 23.6	0.47^b^
Stool calprotectin (mkg/g) at DS	386 ± 66.0	488.9 ± 78.79	0.40^b^

IBD, inflammatory bowel disease; M, male; F, female; CD, Crohn's disease; IBD-U, unclassified colitis; UC, ulcerative colitis; Hb, hemoglobin; ESR, erythrocyte sedimentation rate; CRP, C-reactive protein; Plat, platelets (thrombocytes); PUCAI, Pediatric Ulcerative Colitis Activity Index; PCDAI, Pediatric Crohn's Disease Activity Index.

^a^ Parameters were described as median and range values.

^b^ Parameters were compared by Mann–Whitney *U*-test.

^c^ Fisher's exact test.

^d^ Parameters were compared by Kruskal–Wallis test.

All the above-mentioned data were also comparable in most of the features in terms of IBD patients with and without *MEFV* mutations, and again, no statistically significant difference was found. Particularly, the relationship between the frequency of *MEFV* mutations and IBD relapses in UC patients (22, 59.4%), IBD-U (11; 29.7%), and CD (4; 10.9%) patients, as well as between the presence of severe *M694V* mutation and IBD relapses in patients with coexisting FMF (2.67 ± 0.40) and without it (2.54 ± 0.36), was not significant (*p* = 0.59) (*by Mann–Whitney*). There was no difference found between the presence of *MEFV* mutations and the requirement of immunosuppressants, *p* = 0.5, either. IBD patients of these two groups also had almost the same frequency of extraintestinal symptoms.

### 3.2 Genetic characteristics of IBD patients with MEFV gene mutations

#### 3.2.1 Distribution of MEFV gene mutations depending on IBD type

*MEFV* gene mutations were identified in 37 of 69 (53.6%) IBD patients, and in 33 (47.8%) of them, no *MEFV* mutations were found. Depending on the IBD type, the frequency of *MEFV* mutations was as follows: 22 of 36 (61.1%) UC patients, 11 of 18 (61.1%) IBD-U patients, and 4 of 15 (26.7%) CD patients.

As shown in Table 2, the rate of *MEFV* mutations in UC patients was found to be two times higher than in CD patients (*P* = 0.052; ×2 = 3.74). The *MEFV* mutation carriers are four times more likely to have UC than non-carriers (OR 4.32, 95% CI 1.15–16.28); i.e., the presence of *MEFV* mutation might be considered a risk factor for UC.

**Table 2:** Distribution of MEFV gene mutations depending on IBD type.

Number of IBD patients: 69	Number of IBD patients with *MEFV* mutations depending on IBD type: 37/69 (53.6%)
UC patients, *N* = 36	22 of 36 (61.1%)
CD patients, *N* = 15	4 of 15 (26.7%)
*P* = 0.052; ×2 = 3.74 (OR 4.32, 95% CI 1.15–16.28)	
IBD-U patients, *N* = 18	11 of 18 (61.1%)
CD patients, *N* = 15	4 of 15 (26.7%)
*P* = 0.104; ×2 = 2.65 (OR 4.32, 95% CI 0.98–19.04)	

Meanwhile, despite the same rate of *MEFV* mutations *(and OR value)* in the groups of UC (61.1%; 22 of 36) and IBD-U patients (61.1%; 11 of 18), the comparison of the increased frequency of mutations in the IBD-U group with CD patients was statistically not significant (*P* = 0.104; ×2 = 2.65) (OR 4.32, 95% CI 0.98–19.04), probably due to the small samples sizes.

#### 3.2.2 Distribution of MEFV gene mutations and genotypes among IBD patients

More than half of the IBD patients with *MEFV* mutations (20 of 37; 54.0%) had heterozygous genotypes, 12 (32.4%) had compound heterozygous genotypes, and 5 (13.5%) had homozygotes (Table 3).

**Table 3:** Distribution of *MEFV* gene genotypes among IBD patients.

Distribution of *MEFV* genotypes among IBD patients	Number of IBD patients with *MEFV* mutations *N* = 37
Heterozygous genotypes	20 (54.0%)
Compound heterozygous genotypes	12 (32.4%)
Homozygous genotypes	5 (13.5%)

The M694V mutation was the most common and present in 21 of 37 (56.7%) IBD patients with *MEFV* mutations, mainly in *M694V* compound heterozygous (10/21; 27%) and heterozygous states (8/21; 21.6%). IBD patients with the *MEFV* heterozygous genotype had mostly *M694V* mutation (8 of 17; 47%), less often *V726A* (6 of 17; 35.2%), and rarely *E148Q* (2 of 17) and *M680I* (1 of 17) (Table 4).

**Table 4:** Distribution of *M694V* mutation and M694Vgenotypes among IBD patients.

M694V mutation and genotypes among IBD patients—21/37 (56.7%)	
M694V compound heterozygous genotype (M694V/other mutation)	10 (27%)
M694V heterozygous genotype (M694V/0)	8 (21.6%)
M694V homozygous genotype (M694V/M694V)	3 (8.1%)

### 3.3 Genetic characteristics of UC patients with MEFV mutations and concomitant FMF

MEFV gene mutations were detected in 22 of 36 (61.1%) UC patients, and most of them had the heterozygous (59.0%; 13 of 22) genotype.

A total of 10 of 22 (45.4%) UC patients with *MEFV* mutations had severe *M694V* mutation, mostly (5/10) in heterozygous state (*M694V/0*), 3/10 patients were had *M694V* compound heterozygotes (*M694V/other mutation*), and 2/10 had the *M694V* homozygous genotype *(M694V/M694V).* Moreover, 8 of 22 (36.3%) UC patients with *MEFV* mutations had the *V726A* mutation, 4 of them were in the heterozygous state *(V726A/0),* 2 had compound heterozygotes (*V726A / E148Q),* and 2 had *V726A* homozygotes (*V726A /V726A)*.

The remaining 4 of the 22 (18.2%) UC patients with *MEFV* mutations were heterozygotes for other mutations: 3 patients for *M680I/0* and 1 for *E148Q/0*. Most of the UC patients with *MEFV* mutations had the heterozygous (13 of 22, 59.0%) genotype, mostly for *M694V* (10 of 22, 45.4%) and *V726A* (8 of 22, 36.3%) mutated alleles and rarely for *M680I* (3 of 22, 13.6%).

FMF disease was confirmed in 15 of 36 (41.6%) UC patients, more with compound heterozygous and homozygous genotypes (9/15, 60%) and, again, mainly for the *M694V* mutation (Table 5). Thus, the *M694V* mutation and other *MEFV* mutations were found to be more frequent in UC and IBD-U patients than in CD, but the difference was not significant, *p* = 0.28.

**Table 5:** Distribution *of MEFV* genotypes and concomitant FMF among UC patients.

Distribution of *MEFV* genotypes among UC patients	Number of UC patients with *MEFV* mutations (22/36) (61.1%)	Number of UC patients with concomitant FMF (15/36) (41.6%)
M694V mutation and genotypes: 10/22 (45.4%)		
Heterozygous genotype M694V/0	5	3
Compound heterozygous genotypes M694V/other mutations:M694V/R761H; M694V/V726A; M694V/P369S	3	3
Homozygous genotype M694V/M694V	2	2
V726A mutation and genotypes: 8/22 (36.3%)		
Heterozygous genotypes V726A/0	4	2
Compound heterozygous genotypes V726A/other mutations: V726A/E148Q	2	2
Homozygous genotype V726A/V726A	2	2
Other MEFV mutations and genotypes: 4/22 (18.2%)		
Heterozygous genotypes M680I/0	3	1
E148Q/0	1	

### 3.4 Genetic characteristics of CD patients with MEFV mutations and concomitant FMF

*MEFV* gene mutations were detected in 4 of 15 (26.7%) CD patients, and 3 (20%) of them had the *М694V* mutation and confirmed FMF. Just 1 CD patient was the heterozygous carrier for *E148Q* mutation (without FMF). Thus, FMF disease was confirmed in 3 of 15 (20%) CD patients, with *M694V* mutation in different states (Table 6).

**Table 6:** Distribution of *MEFV* genotypes and concomitant FMF among CD patients.

*MEFV* genotypes among CD patients	Number of CD patients with *MEFV* mutations 4/15 (26.7%)	Number of CD patients with FMF 3/15 (20%)
M694V compound heterozygous genotype: M694V/R761H (1), M694V/M680I (1)	2	2
M694V heterozygous genotype: M694V/0	1	1
Other heterozygous genotypes: E148Q/0	1	–

### 3.5 Genetic characteristics of IBD-U patients with MEFV mutations and concomitant FMF

*MEFV g*ene mutations were revealed in 11 of 18 (61.1%) IBD-U patients. In all, 8 (72.7%) had the *M694V* mutation, mostly (5/8) in the compound heterozygous state (*M694V/V726A* in 3 patients; *M694V/M680I* in 2), 2 (18.1%) were heterozygotes (*M694V/0*), and 1 had the homozygous genotype (*M694V/M694V*). Furthermore, 3 IBD-U patients with *MEFV* (27.2%) were heterozygous carriers for *other mutations* (2 for *V726A/0* and 1 for *E148Q/0)*.

Moreover, 7 of 18 (38.8%) IBD-U patients had concomitant FMF disease, and all of them carried the M694V mutation mostly for the *M694V* compound heterozygous genotypes (5 of 7, 71%). The *V726A* mutation was the second most frequent after the *M694V* mutation (Table 7).

**Table 7:** Distribution of *MEFV* genotypes and concomitant FMF among IBD-U patients.

*MEFV* genotypes among IBD-U patients	Number of IBD-U patients with *MEFV* mutations 11/18 (61.1%	Number of IBD-U patients with FMF 7/18 (38.8%)
M694V mutation: 8/11 (72.7%)		
Compound heterozygous genotype M694V/other mutations: M694V/V726A (3) M694V/M680I (2)	5	5
M694V Heterozygous genotype: M694V/0	2	1
M694V Homozygous genotype: M694V/M694V	1	1
Other heterozygous genotypes: 3/11 (27.2%)		
V726A/0	2	
E148Q/0	1	

Thus, almost half of IBD-U patients with *MEFV* gene mutations (5/11, 45.4%) had heterozygous genotypes and carried mainly *M694V* and *V726A* mutations. FMF disease was confirmed in 7 of 18 (38.8%) IBD-U patients, and all of them had the *M694V* or *V726A* mutation, mostly for compound heterozygous and heterozygous genotypes*.* The majority of UC and IBD-U patients with *MEFV* mutations had heterozygous genotypes (59.0% and 45.4%, respectively), mostly for *M694V* and *V726A* mutated alleles.

### 3.6 Comparison of the distribution of MEFV gene mutations and genotypes among IBD patients with and without FMF

FMF was confirmed in 25 (36.2%) of 69 IBD patients. Depending on the IBD type the frequency of FMF disease was as follows: 15 of 36 UC patients (41.6%), 7 of 18 IBD-U patients (38.8%), and 3 of 15 CD patients (20.0%).

Analysis of the detected *MEFV* mutations in the group of IBD patients *with concomitant FMF* (42 mutations in 25 patients) showed that the *M694* mutation was the most frequent at 21 of 42 (50%), followed by the *V726A* mutation in 12 (28.5%), more rarely *M680I* in 4 (9.5%,), then *E148Q* in 2 (4.7%), *R761H in* 2 (4.7%), and, seldom, *P369S* in 1 (2.3%) (Table 8).

**Table 8:** Distribution of *MEFV* mutations among IBD patients with and without FMF.

Distribution *MEFV* gene mutations among IBD patients		
*MEFV* mutations	With FMF (42 mutations in 25 patients)	Without FMF (11 mutations in 44 patients)
M694V	21 (50%)	3 (27.3%)
V726A	12 (28.5%)	4 (36.4%)
E148Q	2 (4.7%)	3 (27.3%)
M680I	4 (9.5%)	1 (9.1%)
R761H	2 (4.7%)	–
P369S	1 (2.3%)	–

The detection rate of *MEFV* mutations in IBD patients *without FMF* (11 mutations in 44 patients) was as follows: 4 (36.4%) *V726A,* 3 (27.3%) *M694V*, 3 (27.3%) *E148Q,* and 1 (9.1%) *M680I*.

Thus, in IBD patients *with FMF* (25 patients), the *M694V* mutation was found in 21 of 42 (50%) of detected mutations. In the group of IBD patients *without FMF* (44 patients), the *M694V* mutation was revealed in 3 of 11 (27.3%) of *MEFV* mutations.

The highest rate of *MEFV* mutations and FMF disease was recorded in UC patients (61.1% and 41.6%, respectively). However, when comparing the groups of IBD patients with (25 patients) and without FMF (11 patients), we did not find associations either between any *MEFV* mutation and types of IBD or between *MEFV* mutations and concomitant FMF (*P* = 0.28; ×2 = 2.59). In IBD patients with FMF, the M694V mutation was not detected more often than other *MEFV* mutations. That is, in our cohort of IBD patients, the presence of М694V or other MEFV gene mutations did not depend on concomitant FMF.

### 3.7 Genetic characteristics of patients with very early-onset IBD (VEO IBD)

A total of 9 of 25 (36%) IBD patients *with coexisting FMF* developed very early-onset IBD (VEO IBD) during the first 6 years of life. In all, 5 of them (20%) had infantile-onset IBD within the first year of life (4 UC and 1 CD patients). VEO IBD was diagnosed also in 16 of 44 (36.3%) IBD patients *without FMF*, 1 (2%) of whom had infantile-onset IBD. However, there was no significant difference in VEO IBD frequency between these two groups of IBD patients with and without FMF, *p* = 0.82.

Interestingly, all 5 patients (4 UC and 1CD) with infantile-onset IBD and concomitant FMF carried the severe *M694V* mutation in different genotypes: 2 UC patients were homozygotes *(M694V/M694V),* 1 was compound heterozygote (*M694V/V726A),* and 1 heterozygote (*M694V/0).* In addition, 1 CD patient had the compound heterozygous genotype (*M694V/M680I)* (Table 9). They had atypical FMF manifestation with recurrent febrile colitis and/or episodes of diarrhea and abacterial hemocolitis or isolated febrile episodes during the first year of life. Later, typical FMF attacks with aseptic polyserositis (peritonitis, pleuritis, pericarditis) and recurrent arthritis and myalgia developed. All IBD patients with infantile-onset IBD and FMF developed severe courses of both UC and FMF and were partially resistant to conventional IBD treatment. After the diagnosis of FMF and starting maximal dosage of colchicine therapy (0.07–0.08 mg/kg/day), the remission of both diseases was achieved. The patient with infantile-onset CD and concomitant FMF, in addition to the severe enterocolitis, also developed perianal disease and recurrent arthritis. He was fully resistant to colchicine therapy and to immunosuppressive treatment and developed several complications.

**Table 9:** Distribution of *MEFV* genotypes and concomitant FMF among VEO IBD patients.

Distribution of MEFV genotypes and concomitant FMF among VEO IBD patients	Number of patients 25/69
VEO IBD patients *with coexisting FMF*:	9/25 (36%)
Infantile onset: 5 IBD patients (4 UC, 1 CD) 4 4 UC patients:	5/25 (20%)
M694V/M694V	2
M694V/V726A	1
M694V/0	1
1 CD patient:	
M694V/M680I	1
VEO IBD patients without FMF:	16/44 (36.3%)
Infantile-onset IBD-U	1/44 (2%)
VEO IBD patients with and without FMF	*p* = 0.82

## 4 Discussion

According to the data of the National Pediatric Centre for FMF (NPC FMF) of Arabkir MC—ICAH of Armenia, over the last 15 years (2005–2020), there has been a more than six times increase in the total number of FMF-diagnosed children (from 500 to 3250). The annual number of newly diagnosed pediatric FMF cases in NPC FMF is 350 per year. At the same time, according to the hospital-based data of the pediatric gastroenterology service of Arabkir MC—ICAH, over the last 12 years (2008–2020), the total number of pediatric IBD cases increased by three times (from 20 to 69). Among the 3250 FMF pediatric outpatients of the NPC FMF, 0.76% (25) are currently IBD patients, and 0.3% (9) of them have developed VEO IBD. At present, there are no available statistical data in Armenia about the rate of IBD in both adults and children compared to the healthy population.

To the best our knowledge, this is the first study that evaluates the frequency of MEFV gene mutations and FMF disease in 69 Armenian pediatric IBD patients and their influence on the IBD clinical course. The rate of MEFV gene mutations and the frequency of FMF disease were 53.6% and 36.2%, respectively. The highest frequency of *MEFV* mutations and FMF disease was recorded in UC patients (61.1% and 41.6%, respectively), followed by IBD-U patients (61.1% and 38.8%, respectively).

More than half of the IBD patients with *MEFV* mutations had heterozygous genotypes (54.0%), 32.4% had compound heterozygous genotypes, and 13.5% were homozygotes.

A high penetrance of the M694V mutation was the most common and presented among 56.7% of IBD patients. It was found to be more frequent in UC and IBD-U patients, mainly for M694V compound heterozygous and heterozygous genotypes (27% and 21.6%, respectively). As known, the *M694V* mutation is the most frequent FMF-causing mutation in Armenians (50.6%), but, at the same time, among the healthy Armenian population, *M694V* frequency is low at 4.7% (14). Similar data have been presented by authors from Turkey (1, 36).

The rate of *MEFV* mutations in UC patients was found to be twice high as in CD patients (*P* = 0.052**),** and *MEFV* mutation carriers were four times more likely to have UC than non-carriers. Thus, our data suggest that the presence of the *MEFV* mutation might be considered a risk factor for UC patients. Meanwhile, despite the same rate of *MEFV* mutations (and OR value) in the groups of UC (61.1%) and IBD-U patients (61.1%), the comparison of the frequency of these mutations between these patient groups (26.7% %) was statistically not significant (*P* = 0.104), probably due to their small sample sizes.

The majority of UC and IBD-U patients with *MEFV* mutations had heterozygous genotypes (59.0% and 45.4%, respectively), mainly for the *M694V* (27.7%) and *V726A* (22.2%) mutated alleles and more rarely for *M680I (*11.1%) and *E148Q* (2.7%). These data coincide with some other reports, which show that the *M694V* mutation is found not only among IBD patients with a coexisting typical FMF clinical pattern but also among IBD patients with an atypical FMF course or *M694V* mutation carriers. Therefore, in view of the frequent occurrence of FMF in the population at risk, several authors have called for the inclusion of genetic screening for FMF in the investigation of IBD children of Mediterranean ancestry who display unexplained recurrent isolated fever or other atypical FMF manifestations (repeated tonsilitis in early childhood and isolated arthralgia/myalgia, etc.) (20, 40).

The frequency of UC patients with coexisting FMF (41.6%) was significantly higher compared to CD patients with FMF (20%). It should be noted that the frequency of IBD-U patients with FMF was also rather higher (38.8%) than expected but without any significant difference compared to CD patients with FMF.

In the group of IBD patients with concomitant FMF, the *M694V* mutation was also the most frequent and observed in 50% of *MEFV* mutations detected in this group, followed by *V726A* (28.5%) and, more rarely, *M680I* (9.5%). That is, in case of IBD combined with FMF, the *M694V* mutation was detected almost twice more often but without any statistical significance.

However, in the study, when comparing IBD patients *with and without FMF* disease, we did not find associations either between any *MEFV* mutation and types of IBD or FMF coexistence (*P* = 0.28). That is, the presence of the *MEFV* mutation, including severe *М694V* mutation, did not depend on concomitant FMF disease, i.e., the *M694V* mutation in IBD patients with FMF was not detected significantly more often than other *MEFV* mutations.

We have supposed that the *MEFV* gene is not necessarily a susceptibility gene but most likely modifies the course of IBD. This was also indicated in some other studies, which noted that mutations do not have a high impact on the inflammatory response and clinical outcome of the disease (41). Salach S. et al. reported that although 88% of the investigated Egyptian children with IBD carried *MEFV* mutations (the V726A mutation being the commonest), no associations were found between *MEFV* mutations and the phenotypic characteristics of the IBD patients. They suggested that in populations with a high background of the carrier rate of *MEFV* variants, IBD patients should be screened for *MEFV* gene mutations, especially those diagnosed with determinate colitis (42).

Interestingly, in the group of our IBD patients who were MEFV mutation carriers without clinical symptoms of FMF, the *V726A* mutation was also revealed more frequently (36.4%) than *M694V* (27.3%). Moreover, the frequency of the *E148Q* mutation in this group of patients was the same as the *M694V* mutation (27.3%), although the rate of *E148Q i*n healthy Armenian populations is low (3.4%) (8). In a Turkish study, the E148Q mutation was the most common mutation in patients with UC and CD and *M694V* in patients with IC, in contrast to other studies showing that *M694V* was the most common mutation detected in pediatric IBD patients (1, 24, 25, 36)*.* Urgenci et al. studied 597 IBD children from 37 institutions from all over Turkey. They showed that E148Q was the most common mutation in patients with UC and CD and M694V in IC (intermittent colitis): 30,5%, 34,5%, and 47,1%, respectively (41). The E148Q mutation was reported as one of the most common mutations in adult IBD patients (20). Furthermore, a high frequency of some other mutations, particularly *V726A* and *K695R*, was found in other studies (36, 43)

Inflammatory bowel disease (IBD) and familial Mediterranean fever (FMF) are inflammatory diseases with complex interactions among genetic, immune, and environmental factors. Since there are similarities between FMF and IBD, the *MEFV* gene, which is responsible for FMF, has been introduced as a modifier gene for IBD. As reported in many studies, the *MEFV* gene, which frequently causes inflammation, may aggravate the clinical course of UC and CD (17–19, 23, 43). Moreover, some authors suggest that the environment also affects the phenotype of a monogenic disease of the innate inflammatory pathway (44). The concomitant presence of *MEFV* mutations in diseases other than FMF may modify their presence and severity (20, 36, 40, 43). Particularly, recent studies have revealed an increasing spectrum of rare monogenetic diseases, which can present with IBD or IBD-like intestinal inflammation, particularly with VEO IBD. These monogenic disorders also overlap with immunodeficiency and/or autoinflammatory disorders, including hereditary periodic fever syndromes, particularly with FMF (26, 39, 45–47).

In our study,36% of IBD patients with concomitant FMF developed VEO IBD during the first 6 years of life, while 20% of them had infantile-onset IBD within the first year of life. They carried the M694V mutation in different genotypes, had atypical FMF onset and a severe course of both UC/CD and FMF, and were partially resistant to conventional IBD treatment. After the diagnosis of FMF and starting with the colchicine therapy with the maximal dosage, remission of both diseases was achieved. VEO IBD was also diagnosed in 36.3% IBD patients without FMF, and 2% of them had infantile-onset IBD. The VEO IBD was equally frequent in IBD patients with and without FMF (*p* = 0.82). Some authors (24, 26, 28) have confirmed that the cases with unusual early onset of UC in infants and children in ethnically matched populations are alarming, and *MEFV* gene mutations must be evaluated as this association may influence the management of the disease. Particularly, U. Cucinotta et al. concluded that patients with VEO IBD may have a more severe disease course and a poorer response to steroids and anti-TNF-α agents, and they may require more frequent surgical treatment than *P*-IBD patients (30).

A recent study by Y.Furuta et al. (22) has shown that mutations in the MEFV gene may be associated with intestinal inflammation in Behçet's disease. Other authors (48) have proposed the MEFV gene-related enterocolitis concept for some cases diagnosed as IBD-U. Some of them (21) suggested that FMF patients may have accompanied enterocolitis, which could have a clinical course like that of CD.

Several studies have shown an increased rate of *MEFV* mutations and FMF among patients with chronic inflammatory diseases other than IBD, particularly with certain types of vasculitis (IgA vasculitis and PAN-like vasculitis), systemic onset of juvenile idiopathic arthritis (JIA), and spondyloarthropathies (37–39, 41, 49–52)*.* According to the NPC FMF, in Armenian children with FMF, among concurrent pathologies, we found FMF-associated vasculitis (Henoch–Shönlein purpura and protracted febrile myalgia) in 4.3% of patients and, in some cases, higher-than-expected frequencies of JIA (4.7%) and non-amyloid kidney lesions (1.1%) as the first and the only manifestation of the disease, especially when the *M694V* mutation was present (53).

We consider that the limitations of our study are the small sample size of patients, convenient sampling, and conduction in a single center. Therefore, we suppose that further studies are needed.

In this study, despite the small cohort of Armenian pediatric IBD patients, the carrier frequency of *MEFV* gene mutations was rather high at 53.6%, and the frequency of FMF disease among them was 36.2%. The highest rate of them was among UC patients (61.1% and 41.6%, respectively), followed by IBD-U patients, compared to CD. Our data suggest that the presence of the *MEFV* mutation might be considered a risk factor for UC patients. The *M694V* mutation was found to be more frequent in UC and IBD-U patients, more often for the *M694V* heterozygous genotype. The presence of the *MEFV* mutation, including *М694V* mutation, did not depend on concomitant FMF disease. We suppose that the *MEFV* gene is not necessarily a susceptibility gene but most likely modifies the course of IBD, especially UC and U-IBD.

## 5 Conclusion

Considering the high prevalence of FMF in Armenians, *MEFV* genetic testing is recommended for pediatric IBD patients, especially for those with VEO UC and IBD-U, atypical IBD course, or resistance to conventional treatment. They should also be asked for isolated febrile attacks, recurrent arthritis, and family history, even in the absence of typical FMF symptoms, to rule out this monogenic disease. The early diagnosis of FMF and regular colchicine therapy may allow to improve the course of both diseases and prevent complications.

## Data Availability

The original contributions presented in the study are included in the article/supplementary materials, further inquiries can be directed to the corresponding author.
